# Effect of a short course of iron polymaltose on acquisition of malarial parasitaemia in anaemic Indonesian schoolchildren: a randomized trial

**DOI:** 10.1186/s12936-017-1691-5

**Published:** 2017-01-28

**Authors:** Margaretta A. Prasetyani, Quirijn de Mast, Robel Afeworki, Maria M. M. Kaisar, Difa Stefanie, Erliyani Sartono, Taniawati Supali, André J. van der Ven

**Affiliations:** 1 Department of Parasitology, Faculty of Medicine, University of Indonesia, Jakarta, Indonesia; 2 Department of Internal Medicine and Radboud Center for Infectious Diseases, Radboud University Medical Centre, Geert Groote Plein Zuid 8, 6500 HB Nijmegen, The Netherlands; 3 Department of Parasitology, Leiden University Medical Centre, Leiden, The Netherlands

**Keywords:** Iron, Iron polymaltose, Malaria, Plasmodium, Anaemia, *P. falciparum*, *P. vivax*

## Abstract

**Background:**

Concern exists about the safety of iron supplementation given to individuals in malarious areas. The possible unfavourable impact of iron supplementation on malaria might be less when slow-release iron compounds are used instead of ferrous salts, because no toxic non-transferrin bound iron is formed. The aim of this study was to determine the effect of iron supplementation using the slow-release iron compound iron polymaltose (IPM) on the acquisition of malarial parasitaemia.

**Methods:**

A randomized, placebo-controlled trial was performed in schoolchildren aged 5–18 years with mild or moderate anaemia on the Indonesian island Flores. Microscopic malaria-negative children were randomized to receive 8 weeks of IPM (6 mg elemental iron/kg/day) or placebo . The primary outcomes were the occurrence of microscopically detectable malarial parasitaemia at week 4, 8, 12 and 16 after start of treatment and the proportion of participants with real-time (RT) PCR positive malarial parasitaemia at week 16.

**Results:**

294 Children were assigned to the IPM group and 297 to the placebo group. Whereas IPM supplementation failed to increased haemoglobin or ferritin concentrations, the IPM group had a significantly higher rate of occurrence of microscopically detectable parasitaemia [hazard ratio 2.2, 95% C.I. 1.2–4.0; *P* = 0.01]. This higher rate was confined to iron-replete children. At the end of the study, 89% of the children in the IPM group had remained free from microscopically detectable parasitaemia vs 95% of children in the placebo group. The proportion of plasmodial RT-PCR positive children was similar in both groups at week 16 (IPM group 16.6% vs placebo group 14.3%; *P* = 0.47). When analysis was restricted to iron-replete children (serum ferritin ≥30 µg/l), there was a trend for a higher proportion being RT-PCR positive at week 16 in the IPM group compared with the placebo group (20 vs 13.3%; *P* = 0.07). Erythrocyte microcytosis was an independent risk factor for microscopically detectable malarial parasitaemia.

**Conclusions:**

A short course of IPM should be used cautiously in anaemic children in malaria endemic areas, as it has limited efficacy in treating iron deficiency, while it increases the rate of microscopic malarial parasitaemia in those with replete iron stores.

*Trial registration* ISRCTN 83091970. Registered 16 May 2012 (retrospectively registered)

**Electronic supplementary material:**

The online version of this article (doi:10.1186/s12936-017-1691-5) contains supplementary material, which is available to authorized users.

## Background

Iron deficiency is highly prevalent in children in large parts of the world and is associated with anaemia and negative effects on immune function and cognitive development [1]. There has been an ongoing debate on the risk benefit ratio of iron supplementation in malaria endemic regions [2–5], especially following publication of the results from the Pemba trial, which showed increased risk for hospitalization and mortality after untargeted iron supplementation with a low daily iron dose (12.5 mg) among iron-replete children [6].

It is common practice among physicians in many parts of the world to prescribe a short course of iron in a therapeutic dosage to children with anaemia. Whether this practice is associated with an increased risk for malaria is unknown, especially in malaria-endemic areas outside sub-Saharan Africa where malaria caused by *Plasmodium vivax* is more common, malaria transmission is generally less intense and clinical malaria is more frequently seen in older children.

Slow-release iron compounds such as iron polymaltose (IPM) or sodium iron ethylenediaminetetraacetate (NaFeEDTA) are frequently used for iron supplementation instead of ferrous salts in Asian countries. These compounds may have a less unfavourable effect on malaria, as they do not produce toxic non-transferrin bound iron (NTBI) [7–9]. However data on the effectiveness of these compounds in correcting iron deficiency have been conflicting with some studies showing a favourable [10], but others a poor haematologic response [11–13].

The aim of our study was to investigate the effect of a short course of IPM on the risk for microscopic and real-time (RT) PCR positive malarial parasitaemia in anaemic school-aged children with a negative malaria blood slide on Flores Island, Indonesia. Including the number of PCR positive children is relevant as malaria parasite densities in (semi-) immune individuals are frequently below the microscopic detection limit. Secondary objectives were to determine the effects of IPM on haemoglobin (Hb) concentrations and iron status, to determine the influence of baseline iron status and mean cell volume (MCV) on the risk for malarial parasitaemia and the risk of children with submicroscopic malaria to develop microscopic parasitaemia.

## Methods

### Study area

Nangapanda village, Ende district, East Nusa Tenggara Province, Flores Island, Indonesia is a semi-urban village consisting of 18 sub-villages with an estimated population of 22,000 residents. It has a humid climate with an average yearly rainfall of around 1.800 mm and on average 82 rainy days. The rainy season lasts from November until April with a peak in rain during December to March. The primary general health care services are provided in a local Puskesmas (health centre). *Plasmodium falciparum* and *Plasmodium vivax* transmission in this area is stable enough to induce clinical immunity [14]. A population based survey using real-time PCR in the dry season in 2008 showed a prevalence of *P. falciparum*, *P. vivax*, and *Plasmodium malariae* of 14.5, 13.2, and 1.9% respectively [14]. Sub-microscopic parasitaemia was found in more than 80% of all positive cases. Intestinal helminth infections also have a high prevalence in this area [15].

### Trial design, participants and interventions

This study was designed as a prospective double blind placebo-controlled randomized trial. It was conducted between July 2011 and January 2012. Children aged 5–18 years were recruited from twenty-one schools in Nangapanda area. Inclusion criteria were a negative malaria blood slide and mild anaemia, defined as a Hb level 8–11.4 g/dl in children below 7 years old, Hb level 8–11.9 g/dl for girls ≥7 years old and Hb 8–12.9 g/dl for boys ≥7 years old. These cut off levels were based on the 2011 criteria used by the Indonesian national government referral hospital Cipto Mangunkusumo. Exclusion criteria were clinical symptoms suggestive of malaria or any other underlying medical condition.

Enrolled children were treated with the anthelminthic drug albendazole (PT Indofarma Pharmaceutical, Bandung, Indonesia) 400 mg OD for three consecutive days. Subsequently, they were randomized in a 1:1 ratio to receive either iron(III)-hydroxide polymaltose complex (Maltofer^®^, Combiphar, Bandung, Indonesia) or placebo, both taken 6 days a week for 8 consecutive weeks. The iron(III)-hydroxide polymaltose complex tablets of 357 mg contain 100 mg of elemental iron. A weight-based dosage was used aiming at a daily dose of elemental iron of 6 mg/kg daily. Randomization (simple random allocation) was performed using a computer-generated randomization list. The randomization list was created by researchers not involved in this trial, and the study doctors handed out the study medication using this list. Trial participants and laboratory personnel were blinded for treatment assignment; the study doctor was not blinded. The treatment phase started approximately 4 weeks after administration of albendazole and was followed by a follow up phase of another 8 weeks. Teachers handed out the study medication on a daily basis and documented compliance under supervision of the study clinician.

### Follow-up, outcome measures and sample sizes

Children were followed for 96 days (16 weeks) and were reviewed by study clinicians at week 0, 4, 8, 12, and 16 following start of IPM or placebo. These study visits included measurement of body temperature and collection of venous blood, which was used for a full blood count, malaria slide (week 0, 4, 8, 12 and 16) and real-time PCR detection of malarial parasitaemia and serum ferritin concentrations (latter two at week 0 and 16). The primary outcome measure was the detection of microscopically positive malarial parasitaemia at one of the four time points and the number of RT-PCR positive children at week 16. Secondary outcome measurements were the change in Hb and ferritin concentration and MCV value. Malaria slides were examined after the study had ended and results had therefore no clinical consequences for the participating children. With the exception of the full blood count, the other laboratory examinations were also performed after ending of the study. Children with any sign of illness during the study were referred to the Puskesmas.

### Laboratory procedures and definitions

Presence of microscopic parasitaemia was examined by Giemsa staining of both thin and thick blood smears at the Department of Parasitology, University of Indonesia. An aliquot of 200 μl stored whole blood was used for *Plasmodium* DNA isolation, prior to real-time PCR detection using *Plasmodium*-specific primers and species specific probes, as previously described [16]. Haemocytometry was performed using a Sysmex haematological analyzer KX-21 N (Sysmex, Kobe, Japan). A MCV value below 75 fl represented microcytosis. The Mentzer index, which is the ratio between MCV and RBC count, was used as a screening test for thalassaemia using a cut off level <13 being suggestive for thalassaemia [17]. Iron deficiency was defined as ferritin level <30 µg/l, as recommended by the World Health Organization in malaria endemic regions [18].

### Ethics

The study was approved by the ethical committee from the Faculty of Medicine, University of Indonesia (Ref: 96/PT02.FK/ETIK/2010) and all procedures followed were in accordance with the Helsinki Declaration. Parents or caretakers of all children provided written informed consent.

### Statistical analysis

The sample size was calculated to detect a 1.5 hazard ratio (HR) in the IPM group with α = 0.05 and power of 90%. Baseline data are expressed as mean (standard deviation) or median (interquartile range), depending on whether data were normally distributed or as number (%). Differences in the characteristics between the intervention and placebo groups were evaluated by standard statistical methods. Overall cumulative probability to remain free of microscopic parasitaemia during the study period of 16 weeks was assessed by the Kaplan–Meier method and log-rank statistics using an intention to treat approach. Children were censored from the point at which they acquired the first episode of microscopic parasitaemia. Children presenting to the Puskesmas were also censored from the time of presentation. Differences in the proportion of RT-PCR positive children in the IPM and placebo group at week 16 were compared using Chi square tests. Exploratory subgroup analyses were also performed for participants separated on the basis of iron status and baseline MCV value. Unadjusted and adjusted HRs and 95% CI were calculated using Cox proportional hazards model with iron deficiency, age, sex and microcytosis as covariates. The first covariates were pre-specified, the latter (microcytosis) was added later after it was found to be independently associated with outcome. Data were recorded in MS Access (Microsoft Inc.,) and exported for analysis in SPSS 21.0 (SPSS Inc., Chicago, Ill). A *P* value <0.05 signified statistical significance.

## Results

### Baseline characteristics

**Fig. 1:**
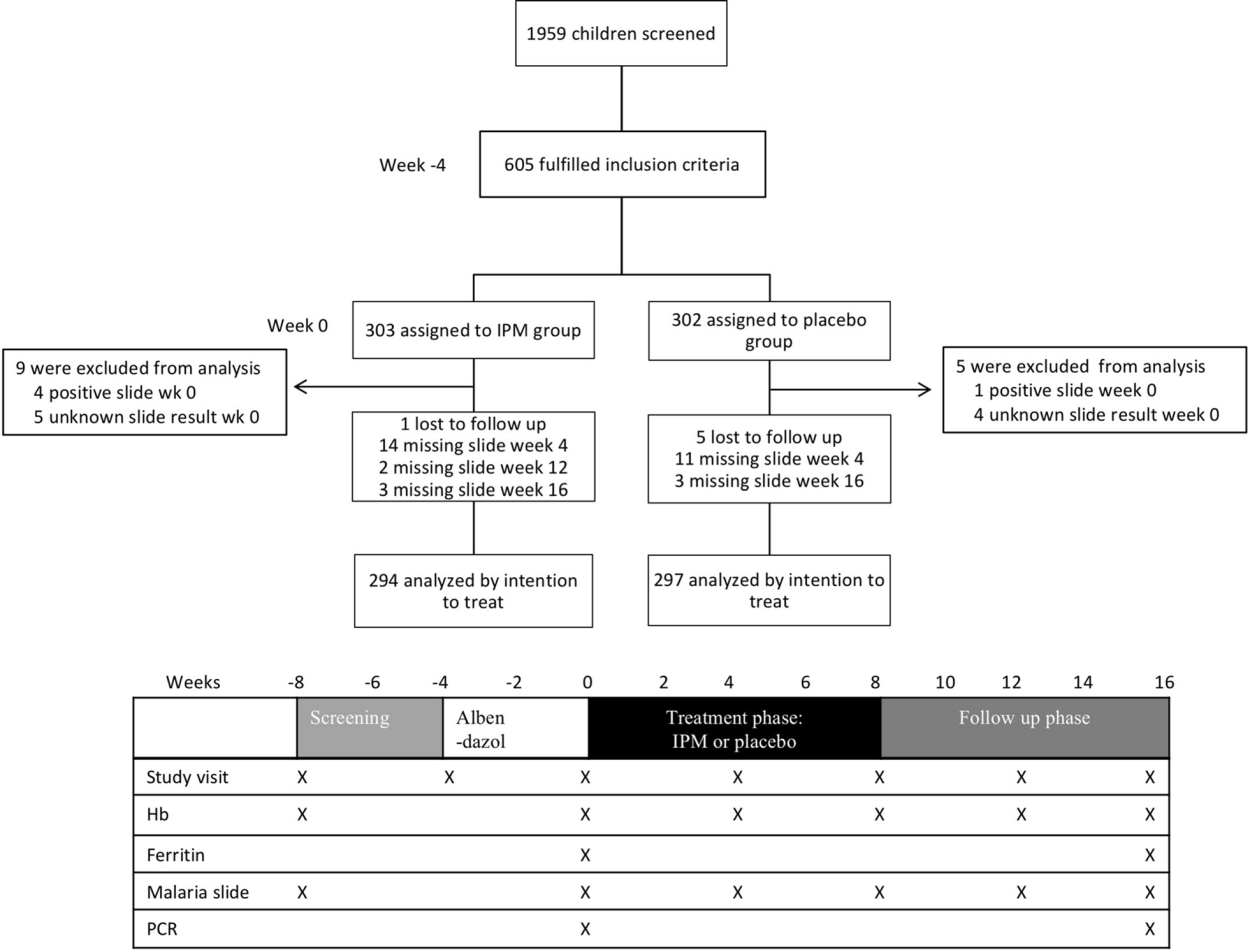
Trial overview and study structure

Figure 1 shows details on the enrolment and follow-up. Data from 591 schoolchildren, of whom 294 were assigned to the IPM group and 297 to the placebo group, were analysed according to intention to treat. The mean age was 10 years with range of 5–18 years and 76% children were male. Baseline characteristics of the children assigned to the two groups were similar (Table 1), including gender, age distribution, weight and values of Hb, ferritin and MCV. Overall, the number of children with a positive PCR for malaria was low (5.8%), with a significantly higher proportion of children with a positive *P. falciparum* PCR in the placebo group than in the iron group (3.7 vs 1.0%; *P* = 0.03). Because of the low prevalence of submicroscopic parasitaemia at baseline, the PCR results for *P. falciparum* and *P. vivax* were combined (referred to as PCR *Plasmodium*) for further analyses. The proportion of children with absolute iron deficiency, defined as a serum ferritin level below 30 µg/l or 12 µg/l, was 26.9 and 8.6%, respectively. In contrast, microcytosis, defined as a MCV value below 75 fl, was present in over half (54.3%) of children. Using the Mentzer index (ratio MCV/RBC count below 13) as a screening test for thalassemia, this condition appeared common with an estimated overall prevalence of 23.0%.

**Table 1:** Baseline characteristics of the 591 children from Flores island, Indonesia

Variable^a^	IPM group	Placebo group
Number	294	297
Gender, male, n (%)	225 (77)	224 (75)
Age, years, mean ± SD	10.2 ± 2.8	10.5 ± 3.0
Baseline age 5–11, n (%)	206 (70)	190 (64)
Baseline age 12–15, n (%)	59 (20)	72 (24)
Baseline age 16–18, n (%)	29 (10)	35 (12)
Weight, kg, mean ± SD	24.8 ± 8.1	26 ± 9.7
PCR *Plasmodium*, n (%)	16 (5.4)	18 (6.1)
PCR *P. falciparum*, n (%)	3 (1.0)	11 (3.7)
PCR *P. vivax*, n (%)	13 (4.4)	7 (2.4)
Haemoglobin concentration, g/dl, mean ± SD	12.0 ± 0.8	12.0 ± 0.9
Haematocrit, mean ± SD	37.4 ± 3.1	37.7 ± 2.5
Mean corpuscular volume (MCV), fl, mean ± SD	74.2 ± 5.5	73.7 ± 5.7
MCV < 75 fl, n (%)	147 (52)	164 (57)
MCV ≥ 75 fl, n (%)	147 (48)	132 (43)
Ferritin concentration, µg/l, median (IQR)	43.5 (28.3–64.8)	40.4 (26.6–64.2)
Number with ferritin < 30 µg/l, n (%)	77 (26)	82 (28)
With MCV < 75 fl, n (%)	39 (13)	52 (18)
With MCV ≥ 75 fl, n (%)	38 (13)	29 (10)
Number with ferritin < 12 µg/l, n (%)	18 (6)	33 (11)
With MCV < 75 fl, n (%)	6 (2)	21 (7)
With MCV > 75 fl, n (%)	12 (4)	11 (4)
Suspected thalassaemia, n (%)^b^	62 (21)	74 (25)

^a^Differences in distribution of baseline characteristics were analysed using the Chi square test or Student *t* test using log transformed data. There were no statistically significant differences between except in number of number with positive PCR *Plasmodium falciparum* (P = 0.03), number of children with ferritin level < 12 µg/l) (P = 0.03) and number of children with serum ferritin level < 12 µg/l and microcytosis (P = 0.03)

^b^Suspected thalassemia was defined as Mentzer index (MI) < 13, the ratio of (MCV/RBC)

*IPM* iron polymaltose, *IQR* interquartile range, *PCR* polymerase chain reaction

A total of 13 children (10 from IPM group and 3 placebo group) were seen in between the regular study visits at the local health centre because of different clinical symptoms (Additional file 1). They were not reviewed in a systematic way. Fever was among the symptoms in 4 children in the IPM group and one child in the placebo group. Because a final diagnosis was not established in these children and malaria diagnostics were not performed, they were censored from the time-point they presented to the local health centre.

### Effect of IPM on Hb and ferritin concentrations

Table 2 shows data of Hb, ferritin and MCV at week 16 and the change in these parameters from baseline. Changes in values of Hb, serum ferritin and MCV were small and similar in both groups. Even in the subgroup of children with absolute iron deficiency at baseline, the increase in serum ferritin was small and similar between the groups. There was also no difference in the proportion of children remaining iron deficient at week 16 between the IPM and placebo groups.

**Table 2:** Haemoglobin, ferritin and MCV values at end of study

Variable	IPM group	Placebo group
Haemoglobin level available, n	290	288
Haemoglobin level, g/dl, mean ± SD	12.1 ± 1.0	12.0 ± 1.1
∆ Haemoglobin, g/dl, mean ± SD	0.09 ± .91	0.08 ± .85
Ferritin level available, n	277	276
Ferritin level, µg/l, median (IQR)	47.7 (30.8–70.4)	45.2 (28.8–65.8)
∆ Ferritin, µg/l, median (IQR)	3.0 (−9.9 to 17.7)	1.3 (−9.4 to 14.6)
Children with iron deficiency at baseline, n^a^	77	82
Children remaining iron deficient at wk 16, n (%)	43 (55.8)	53 (64.6)
∆ Ferritin, µg/l, median (IQR)	6.1 (2.6–17.5)	5.9 (−0.1 to 14.5)
Mean corpuscular volume (MCV) available, n	290	288
MCV, fl, mean ± SD	74.7 ± 5.4	74 ± 5.6
∆ MCV, fl, mean ± SD	0.5 ± 2.2	0.4 ± 1.9
Children with microcytosis at baseline, n^b^	147	164
Persistent microcytosis at wk 16, n (%)	133 (87.5)	148 (87.6)
∆ MCV, fl, mean ± SD	1.1 (0.7–1.5)	0.7 (0.4–1.1)

There were no statistical significant differences in any parameter between the groups

^a^Iron deficiency defined as serum ferritin concentration < 30 μg/l

^b^Microcytosis defined as MCV ≤ 75 fl

∆ = Mean or median change between baseline and week 16

### Effect of IPM on microscopically detectable parasitaemia

**Fig. 2:**
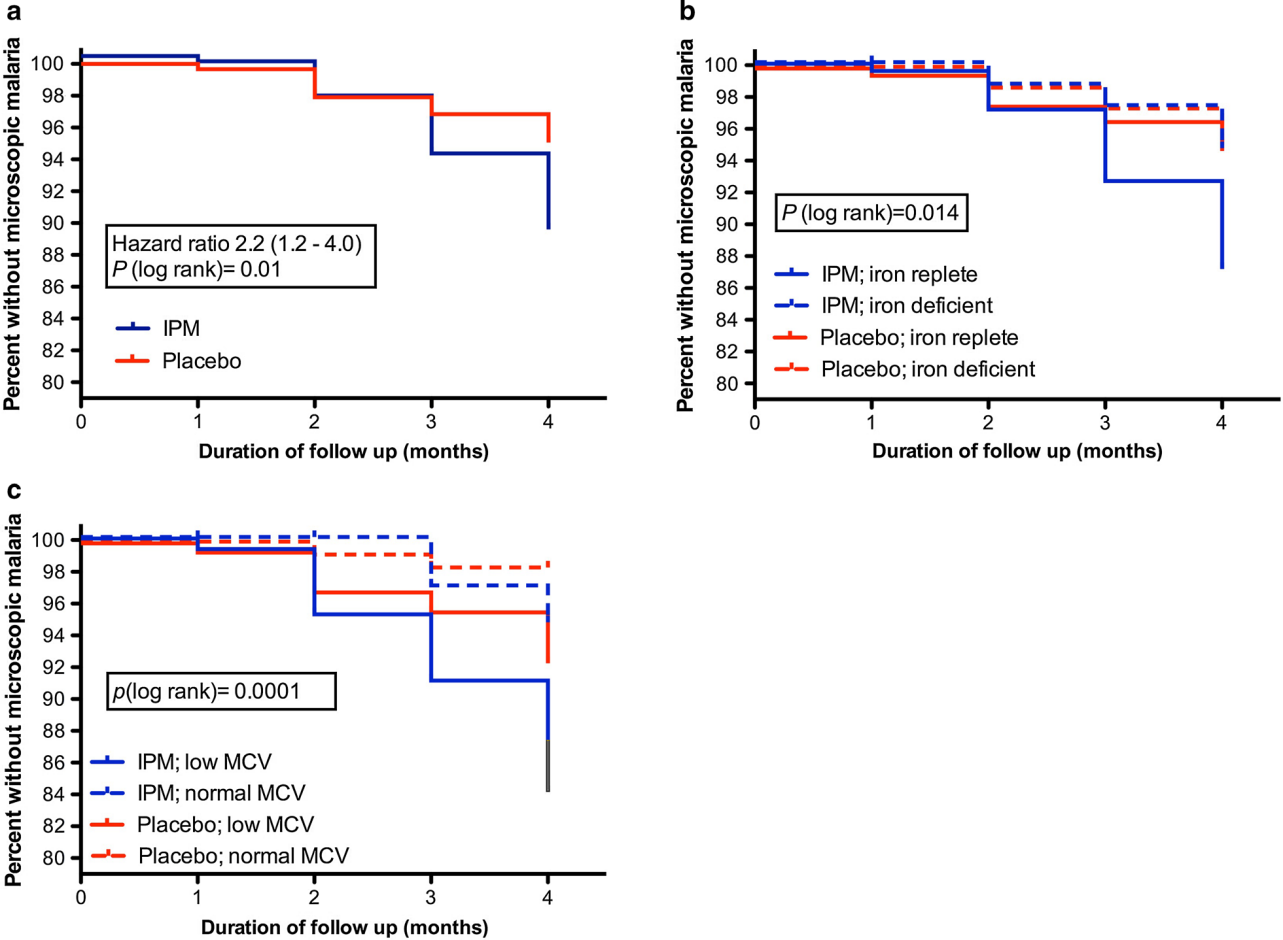
Kaplan-Meier plots for the first episode of microscopic malaria in the whole group (**a**) and aggregated by iron status (**b**) and mean corpuscular volume (MCV) (**c**). Statistical comparison was performed with long-rank test with Bonferroni correction. *IPM* iron polymaltose

Figure 2 shows that children assigned to the IPM group had a significantly higher rate of development of microscopically detectable parasitaemia than those assigned to the placebo group (unadjusted hazard ratio (HR) 2.2, 95% CI 1.2–4.0; *P* = 0.01). The cumulative number of children developing microscopic positive parasitaemia between week 0 and 16 was 29 (19 *P. falciparum*, 10 *P. vivax*) in the IPM group and 14 (8 *P. falciparum*, 6 *P. vivax*) in the placebo group. At the end of the study, 89% of the children in the IPM group had remained free from microscopically detectable parasitaemia vs 95% of children in the placebo group. Seven children had fever (≥38 °C) at one of the four regular study visits time; three of these children had a positive malaria slide on the same time point. Exploratory analysis showed that when children were stratified by iron status, the increased rate of microscopic malaria in the IPM group was due to the increased rate in children who were iron replete (serum ferritin ≥30 μg/l) at baseline (Fig. 2b). Iron deficient children in the IPM group had a similar incidence of microscopically detectable parasitaemia as children in the placebo group (95% of children free of parasitaemia during study). When children were stratified by MCV category, those with microcytosis (MCV value <75 fl) had the highest rate of developing microscopically detectable parasitaemia (Fig. 2c). Univariate and multivariate adjusted hazard ratio for the first episode of microscopic malaria were also calculated using Cox regression with treatment arm, baseline iron status, baseline MCV value and age as variables (Table 3). Assignment to the iron group and microcytosis were independently associated with an increased rate of microscopic malaria, while baseline iron status and age were not.

**Table 3:** Univariate and multivariate adjusted hazard ratio for the first episode of microscopic malarial parasitaemia

Variable	Univariate		Multivariate	
	HR (95% CI)	*P* value	HR (95% CI)	*P* value
Iron treatment	2.2 (1.2–4.2)	0.013	2.3 (1.2–4.3)	0.012
Microcytosis	3.3 (1.6–7.1)	0.001	3.4 (1.7–7.1)	0.001
Iron deficiency	0.6 (0.3–1.3)	0.18	0.6 (0.3–1.4)	0.22
Age	0.9 (0.8–1.0)	0.22	1.0 (0.9–1.1)	0.63
Female sex	1.3 (0.7–2.5)	0.43	1.7 (0.9–3.2)	0.13

Microcytosis defined as MCV value < 75 fl; iron deficiency as ferritin concentration < 30 µg/l. Age was included as continuous variable

*HR* hazard ratio, *CI* confidence interval

### The effect of IPM on PCR-based detection of malaria

From the children with submicroscopic malaria at baseline (16 in the iron group and 18 in the placebo group), only 3 developed microscopically detectable parasitaemia during follow up (2 in the IPM group and 1 in the placebo group). At the end of the study, 5 children in the IPM group and 7 in the placebo group had remained PCR positive for *Plasmodium*. Results of the *Plasmodium* PCR at week 16 were available in 278 of children in the IPM group and 286 of children in the placebo group and are summarized in Table 4. A positive PCR result was found in 46 (16.6%; 23 *P. falciparum*, 18 *P. vivax*, 5 mixed infection) of children in the IPM group and in 41 (14.3%; 23 *P. falciparum*, 14 *P. vivax*, 4 mixed infection) in the placebo arm (*P* = 0.47). Analysing only iron-replete children, we found that 41 (20.0%; 20 *P. falciparum*, 16 *P. vivax*, 5 mixed infection) out of 205 children in the IPM group and 28 (13.3%; 15 *P. falciparum*, 10 *P. vivax*, 3 mixed infection) out of 210 children in the placebo group (P = 0.07) were PCR *Plasmodium* positive. Overall, a higher proportion of children with microcytosis at baseline had a positive *Plasmodium* PCR at week 16 compared with children with a normal MCV value (19.3 vs 9.3%; *P* = 0.001). However, in the subgroups of children with or without microcytosis, the proportion of *Plasmodium* PCR positive children was similar in the IPM and placebo arms.

**Table 4:** Number (proportion) of participants with a positive Plasmodium RT-PCR at week 16

	IPM group No. (%)	Placebo group No. (%)	*P* value
Overall	46/278 (16.6)	41/286 (14.3)	0.47
Iron status baseline			
Iron replete	41/205 (20.0)	28/210 (13.3)	0.07
Iron deficient	5/73 (12.6)	13/76 (17.1)	0.06
MCV baseline			
Microcytosis	31/143 (21.7)	31/161 (19.3)	0.60
No microcytosis	15/135 (11.1)	10/124 (8.1)	0.41

## Discussion

The main findings of this study are that a short course (8 weeks) of therapeutic iron supplementation using the slow release iron compound IPM was ineffective in raising Hb or ferritin levels in anaemic Indonesian schoolchildren, whereas it did increase the risk for microscopically detectable plasmodial parasitaemia, as has previously been shown for ferrous salts in a fortification [6] and therapeutic dose [19]. Similar to findings from the Pemba study [6], this increased risk was restricted to children who were iron-replete. Furthermore, in our study population, a low MCV value was independently associated with an increased incidence of microscopically detectable parasitaemia.

Worldwide, the most common method of screening individuals for iron deficiency involves measuring haemoglobin or haematocrit. It should be taken into account, however, that iron deficiency anaemia only represents the end of the spectrum of iron deficiency. Recent iron supplementation studies in malaria areas were generally performed in children less than 5 years old from sub-Saharan Africa in whom anaemia and iron deficiency were common. The Indonesian children enrolled in our study were older, and although anaemia was also relatively common in the study area, it was mostly mild with relatively high iron stores (based on ferritin levels). In many low-resource areas, measuring iron status indicators such as ferritin is neither affordable nor logistically feasible. Instead of targeted iron supplementation based on iron status, supplementation using safer iron compounds that do not increase the risk for malaria would offer a valuable alternative. Unfortunately, the hypothesis that IPM would represent such an alternative, because it does not lead to the formation of NTBI in contrast to ferrous salts [9, 20], proved incorrect. In addition, the data highlight the importance of malaria prevention measures and prompt diagnostics and treatment of malaria when children are given iron supplements in malaria-endemic regions.

IPM also had poor effectiveness in treating iron deficiency. This finding does not stand alone, as multiple studies have previously shown reduced or even absent effectiveness of IPM in correcting iron deficiency [11–13, 21]. Iron absorption studies have also shown a significant lower iron bioavailability of IPM compared with ferrous salts [22, 23], especially when taken on an empty stomach. This poor bioavailability was the reason that a high iron dose of 6 mg/kg body weight was used in our study, like in previous iron supplementation studies in children [12]. Other reasons for the low efficacy of IPM include the fact that moderate anaemia and absolute iron deficiency were relatively rare, the fact that albendazole was given at enrolment and the possible confounding effects of inflammation. The latter impairs iron absorption through the effects of the iron regulatory hormone hepcidin. It is possible that a proportion of children experienced a period of asymptomatic malarial parasitaemia during the course of the study. Previous studies have shown that asymptomatic parasitaemia increases hepcidin concentrations and that treatment with anti-malarials improves iron absorption [24, 25]. Nonetheless, the confounding effects of malaria-associated inflammation in the present study appeared limited as children with patent parasitaemia were excluded from enrolment and the prevalence of subpatent parasitaemia was low.

The mechanisms responsible for the higher incidence of malarial parasitaemia in those assigned to IPM remain speculative, especially since a clear effect of IPM on iron status was absent. It is also not easy to reconcile this finding with the fact that the increased malaria risk was confined to iron replete children. Moreover, only a minority of children with submicroscopic malaria at enrollment developed microscopically detectable parasitaemia and this risk was similar in the IPM and placebo groups. As reviewed recently, many questions still remain on how exactly iron deficiency protects against malaria and how iron supplementation may increase the risk for malaria [26]. Direct use of iron by parasites, iron-related changes in erythropoiesis and the immune and vascular effects of iron deficiency and iron supplementation may all be involved [26]. However, other factors may also play a role. After intake, only a small percentage of iron is absorbed in the small intestine and most of the iron passes the colon. Recent studies indicate that iron can induce overgrowth of enteric pathogens and bowel inflammation [27–29] and an association between the composition of the stool microbiota and the prospective risk for malaria was recently shown [30]. These enteric effects of iron on malaria pathogenesis certainly warrant further study.

Whereas the Pemba trial showed an adverse effect of iron supplementation on malaria [6], another recent trial in Ghana, in an area where insecticide-treated bed nets and malaria treatment were readily available, showed a lower malaria incidence in children assigned to an iron containing micronutrient group, although the number of hospital admissions in this group was higher [31]. The recent WHO guideline on daily iron supplementation in infants and children also concludes that in malaria-endemic areas, iron supplementation does not increase the risk of clinical malaria or death when regular malaria-surveillance and treatment services are provided [32]. In the study area in Indonesia where this study was conducted, *P. vivax* malaria is also common, and although the number of *P. vivax* cases was insufficient to analyse separately, its incidence appeared higher in the IPM group. This is consistent with two previous studies in children and pregnant women in which iron supplementation was associated with increased *P. vivax* burden [33, 34].

The primary outcome in this study was microscopically or RT-PCR detectable plasmodial parasitaemia. It lacked sufficient power to examine clinical endpoints as the development of febrile malaria was rare and only few clinical events were noted. Only three children with a positive malaria slide had a concurrent fever at the same time point, suggesting that most of the children had asymptomatic parasitaemia. The observation that febrile malaria appeared rare is explained by the fact that the study was performed in semi-immune children from a moderate malaria transmission area. It can also not be excluded that the thirteen children who visited the local health centre had clinical malaria. However, fever was only reported by five of these children and many had symptoms suggestive for an alternative illness than malaria.

The data also showed that microcytosis was independently associated with the development of microscopic malaria. Although MCV has been included as a parameter in iron studies in malaria endemic regions [35–37], it was not analysed as an independent risk-factor for malaria. Opposite to the present findings, one might assume that microscopic anaemia identifies children at lower risk for malaria during iron treatment, as microcytosis is commonly seen in iron deficiency and thalassaemia, conditions both considered to be associated with protection against clinical malaria. Unfortunately, it was not feasible to perform haemoglobin electrophoresis to diagnose thalassaemia, but based on the Mentzer index, thalassaemia was suspected in around 50% of children with microcytosis. Because MCV is part of the Mentzer index, it was also not feasible to assess this index as an independent risk factor for parasitaemia. *Plasmodium vivax* has a strong predilection for young erythrocytes, particularly reticulocytes, whereas *P. falciparum* also invades older and therefore more microcytic cells [38]. Recent in vitro studies, however, suggested that *P. falciparum* favours invasion and replication in young erythrocytes as well [26]. One may speculate that infection of young and larger erythrocytes results in premature senescence and more rapid clearance from the circulation while senescence of infected microcytic cells is delayed.

This study has several limitations. First, the number of submicroscopic and microscopic malaria cases at baseline and during the study was lower than anticipated limiting the study power and the ability to analyse clinical malaria cases and *P. vivax* separately. Malaria prevalence studies, conducted in the years prior to start of this study, had shown a prevalence of parasitaemia of 26.4% [14]. Second, whereas laboratory personnel who performed the malaria diagnostics were blinded for treatment allocation, the study doctor was not. Third, NTBI levels were also not available, partly because its correct measurement is technically challenging. Fourth, children who fell ill were assessed in the local health centre and were not assessed by the study team. As mentioned earlier, it is therefore unknown whether malaria was the cause of their illness. As 10 of these children were assigned to the IPM arm and 3 to the placebo arm, it is unlikely that the conclusions from our study would change in case malaria was indeed the cause of the illness. Finally, ferritin levels at baseline were not corrected for inflammation, although it is unlikely that inflammation was common in the participants, as those with microscopically parasitaemia were excluded.

## Conclusions

A short course of IPM had limited efficacy in treating iron deficiency, but still increased the rate of occurrence of microscopic malarial parasitaemia. IPM is widely used in Asia, but the present findings add to the evidence that its use in anaemic children should be discouraged.

## Additional file

**Additional file 1.** Clinical data from children who visited the health centre (Puskesmas) during the study.

## Abbreviations

Hb: haemoglobin
HR: hazard ratio
IPM: iron polymaltose
MCV: mean cell volume
NTBI: non-transferrin bound iron
