# Supplementary material for: Effect of a short course of iron polymaltose on acquisition of malarial parasitaemia in anaemic Indonesian schoolchildren: a randomized trial
Source: Malar J. 2017 Jan 28;16:50. doi: 10.1186/s12936-017-1691-5 (PMC5273788; doi:10.1186/s12936-017-1691-5)
Supplement: Supplementary file 1 — Additional file 1. Clinical data from children who visited the health centre (Puskesmas) during the study. [file 12936_2017_1691_MOESM1_ESM.docx]

Supplemental material

**Supplemental table 1.** Clinical data from children who visited the health centre (Puskesmas) during the study.

| **Date of visit** | **Iron or placebo** | **Primary symptoms** | **Temp. (^o^C)** | **Other symptoms** |
| --- | --- | --- | --- | --- |
| 20/09/2011 | Iron | Skin blisters (varicella) (2 days) | 36.8 | Fever, nausea, chest pain, skin infection |
| 22/09/2011 | Iron | Vomiting, diarrhea (1 day) | 36.0 | Nausea, skin infection |
| 06/10/2011 | Iron | Headache (1 week) | 36.8 | Shivering, arthralgia |
| 07/10/2011 | Placebo | Fever (1 day) | 36.4 | Shivering, nausea |
| 10/10/2011 | Iron | Toothache, headache (2 days) | NA | Night sweats, headache |
| 01/11/2011 | Iron | Fever, vomiting (5 days) | NA | Night sweats, headache, nausea, cough, running nose |
| 15/10/2011 | Iron | Swollen cheek (3 days) | 36.0 | Headache, cough, running nose |
| 17/10/2011 | Iron | Fever (2 days) | 37.8 |  |
| 20/10/2011 | Placebo | Heartburn, headache | NA |  |
| 21/10/2011 | Iron | Diarrhea, cough (1 day) | NA | Headache, running nose |
| 24/10/2011 | Placebo | Weakness, headache | 36.5 |  |
| 24/10/2011 | Iron | Abdominal pain (1 day) | NA | Heartburn |
| 08/11/2011 | Iron | Fever, cough (2 days) | 37.6 |  |

NA, not available
